# A Mitochondria-Targeted Nitroxide Radical Mitigates Radiation-Induced Liver Injury by Attenuating Oxidative Stress and Preserving Mitochondrial Function

**DOI:** 10.3390/antiox15060780

**Published:** 2026-06-22

**Authors:** Miaomiao Li, Xiaojun Deng, Haibo Wang

**Affiliations:** 1 Department of Chemistry, School of Pharmacy, The Fourth Military Medical University, Xi’an 710032, China; 223040012830@email.sntcm.edu.cn (M.L.); dengxiaojun@fmmu.edu.cn (X.D.); 2 Department of Pharmacy, Shaanxi University of Chinese Medicine, Xianyang 712046, China

**Keywords:** radioprotector, mitochondria-targeted antioxidant, radiation-induced liver injury, oxidative stress, selective protection

## Abstract

Radiation-induced liver injury (RILI) is a major complication of abdominal radiotherapy, originating from mitochondrial oxidative stress, and effective radioprotectants are lacking. We designed an antioxidant intended to target mitochondria, TPP-C6-NIT, by conjugating a triphenylphosphonium cation to an imidazole nitroxide radical. Its protective effects were evaluated through in vitro assays, studies on irradiated L-02 and Huh-7 cells, a mouse model of whole-body irradiation, combined with metabolomics, molecular docking, and assessments of mitochondrial function, apoptosis, and inflammation. TPP-C6-NIT exhibited potent radical scavenging activity in vitro. In L-02 cells, it reduced oxidative stress, preserved mitochondrial function (membrane potential, ATP, respiratory capacity), and improved viability. In mice, pretreatment with TPP-C6-NIT significantly improved survival, alleviated liver injury (reduced serum ALT/AST and histopathological damage), and suppressed systemic inflammation. Mechanistic exploration suggested TPP-C6-NIT treatment was associated with increased Nrf2/GPX4 expression and reversal of lipid metabolic changes. Notably, TPP-C6-NIT did not confer significant protection in Huh-7 cells, indicating selective cytoprotection. By reducing oxidative stress and preserving mitochondrial function, TPP-C6-NIT demonstrates potent protection against radiation-induced liver injury in a whole-body irradiation mouse model, presenting a promising candidate for further development.

## 1. Introduction

Radiation-induced liver injury (RILI) is a major dose-limiting complication of abdominal and hepatic tumor radiotherapy, severely impacting therapeutic efficacy and patient prognosis [1,2,3]. The core pathophysiological process of RILI initiates from ionizing radiation-induced mitochondrial oxidative stress. Excessive reactive oxygen species (ROS), generated directly or indirectly by radiation, can attack the mitochondrial electron transport chain, triggering lipid peroxidation, collapse of membrane potential, and energy metabolism failure. This cascade subsequently leads to hepatocyte apoptosis, necrosis, and persistent inflammation, ultimately driving tissue fibrosis and organ function loss [4,5]. Therefore, targeting and protecting mitochondria to curb the oxidative stress cascade at its source represents one of the most promising strategies for preventing and treating RILI [6].

However, the development of ideal radioprotective agents faces two major challenges: protective efficacy and selectivity. Conventional antioxidants (e.g., amifostine) often lack target specificity, making it difficult to achieve effective concentrations at the core damage site (mitochondria), and their systemic side effects limit clinical application [7,8]. More critically, an ideal protectant should safeguard normal tissues without compromising the therapeutic efficacy of radiotherapy against tumor cells. This “selective” property is rarely reported among existing agents.

Nitroxide radicals are a class of catalytic antioxidants characterized by a stable single-electron structure. They not only directly quench various ROS but also mimic superoxide dismutase activity through redox cycling, demonstrating unique antioxidant potential [9,10]. To overcome their non-targeting limitations, mitochondrial-targeted delivery strategies have emerged [11]. Among these, lipophilic cations (e.g., triphenylphosphonium, TPP^+^) can accumulate across membranes by leveraging the negative-inside mitochondrial membrane potential (ΔΨ_m_), and have been successfully utilized to construct various “targeting-effector” conjugate molecules [12,13]. We hypothesized that combining the targeting capability of TPP^+^ with the potent antioxidant activity of nitroxide radicals could lead to the design of novel protective molecules capable of actively “navigating” to the frontline of mitochondrial damage.

In this study, we designed and synthesized a novel mitochondria-targeted antioxidant, TPP-C6-NIT. This molecule covalently links the TPP^+^ targeting moiety to an imidazole nitroxide radical (NIT) antioxidant unit via a flexible alkyl linker. We aimed to test the hypothesis that TPP-C6-NIT, by targeting mitochondria, can scavenge ROS at their source, alleviate radiation-induced oxidative stress, preserve mitochondrial function, and maintain cellular energy homeostasis, thereby conferring protection to normal hepatocytes and liver tissue. Concurrently, given the frequent dysregulation of redox homeostasis in tumor cells, this protective effect might be selective, i.e., not interfering with the radiosensitivity of hepatocellular carcinoma cells.

To this end, we first assessed the free radical scavenging capacity of TPP-C6-NIT in vitro. Secondly, at the cellular level, we evaluated its protective effects against radiation-induced oxidative stress and mitochondrial dysfunction (membrane potential, ATP production, respiratory capacity) using irradiated normal L-02 hepatocytes, while preliminarily exploring its selectivity using Huh-7 hepatocellular carcinoma cells as a control. Subsequently, in a mouse model of whole-body irradiation, we assessed the protective effects of TPP-C6-NIT on survival rate, liver function (serum ALT/AST), and liver histopathological damage, and analyzed its effects on systemic inflammation. Additionally, we explored molecular features associated with TPP-C6-NIT treatment, including Nrf2/GPX4 expression and lipid metabolism (via metabolomics), and performed molecular docking simulations to generate hypotheses for future mechanistic studies.

This study provides evidence that TPP-C6-NIT, by reducing oxidative stress and preserving mitochondrial function, offers efficient protection against radiation-induced liver injury in a whole-body irradiation mouse model and shows minimal protection in Huh-7 cells, presenting a lead compound for the development of organelle-targeted radioprotectants.

## 2. Materials and Methods

### 2.1. Compounds, Reagents, and Cells

The novel mitochondria-targeted antioxidant TPP-C6-NIT was designed and synthesized in our laboratory, with a purity of >98% confirmed by HPLC and HRMS. The positive control drug amifostine (WR-2721) was purchased from Shanghai Jizhi Biochemical Technology Co., Ltd. (Shanghai, China). Assay kits for superoxide dismutase (SOD), glutathione peroxidase (GSH-Px), malondialdehyde (MDA), and glutathione (GSH/GSSG), as well as ELISA kits, were obtained from the Nanjing Jiancheng Bioengineering Institute (Nanjing, China). The CCK-8 reagent, the reactive oxygen species (ROS) fluorescent probe DCFH-DA, and the mitochondrial membrane potential (ΔΨ_m_) probe JC-1 were purchased from Beyotime Biotechnology (Shanghai, China). 1,1-Diphenyl-2-picrylhydrazyl (DPPH) and 2,2′-azino-bis(3-ethylbenzothiazoline-6-sulfonic acid) (ABTS) were purchased from Shanghai Hualan Chemical Technology Co., Ltd. (Shanghai, China). The TUNEL assay kit was also obtained from Servicebio (Wuhan, China).

Human normal hepatocytes (L-02 cells) and human hepatocellular carcinoma cells (Huh-7 cells) were purchased from Beina Chuanglian Biotechnology Co., Ltd. (Xinyang, China). L-02 cells were cultured in RPMI-1640 medium supplemented with 20% fetal bovine serum (FBS), while Huh-7 cells were cultured in DMEM medium supplemented with 10% FBS. All cells were maintained at 37 °C in a humidified atmosphere containing 5% CO_2_.

### 2.2. In Vitro Antioxidant Activity Assay

The in vitro antioxidant capacity of TPP-C6-NIT was evaluated using 1,1-diphenyl-2-picrylhydrazyl (DPPH) and 2,2′-azino-bis(3-ethylbenzothiazoline-6-sulfonic acid) (ABTS) radical scavenging assays [14].

DPPH Radical Scavenging Assay. TPP-C6-NIT solutions (25, 50, 75 μg/mL) were mixed with an equal volume of 100 μM DPPH in anhydrous ethanol. The mixtures were incubated in the dark at room temperature for 30 min. Absorbance was measured at 517 nm using a microplate reader (Thermo Fisher Scientific, Waltham, MA, USA). The radical scavenging rate (%) was calculated as: Scavenging Rate (%) = [ (A_0_ − A_1_)/A_0_] × 100%, where A_0_ is the absorbance of the DPPH solution without the test compound, and A_1_ is the absorbance of the DPPH solution with the test compound.

ABTS^+^• Radical Scavenging Assay. A 7 mM ABTS aqueous solution was mixed with an equal volume of 2.45 mM potassium persulfate solution and allowed to react in the dark at room temperature for 16 h to generate the ABTS^+^• stock solution. Prior to use, the stock solution was diluted with phosphate-buffered saline (PBS) until its absorbance at 734 nm reached 0.70 ± 0.02. Different concentrations of the test compound solution were mixed with an equal volume of the ABTS^+^• working solution and incubated in the dark at room temperature for 10 min. Absorbance was measured at 734 nm, and the scavenging rate was calculated using the formula above. All assays were independently performed in triplicate.

### 2.3. Evaluation of Radioprotection, Oxidative Stress, and Preliminary Mechanisms at the Cellular Level

#### 2.3.1. Cell Viability and Radioprotective Effect

The acute radioprotective effect of TPP-C6-NIT was assessed using the CCK-8 assay. Briefly, L-02 or Huh-7 cells in the logarithmic growth phase were seeded into 96-well plates at a density of 5 × 10^3^ cells per well. After attachment, the medium was replaced with fresh complete medium containing different concentrations of TPP-C6-NIT (0.15, 0.3, 0.6 μM) or the positive control WR-2721 (1.5 μM) for a 1 h pretreatment. Subsequently, the cells were exposed to a single dose of X-ray irradiation at 0, 2, 4, 6, or 8 Gy (dose rate: 1.414 Gy/min). After irradiation, the medium was replaced with fresh drug-free medium, and the cells were cultured for an additional 72 h. Then, 10 μL of CCK-8 solution was added to each well, and after incubation for 1–4 h, the absorbance was measured at 450 nm.

The optimal concentrations of TPP-C6-NIT for in vitro use (0.15–0.6 μM) were determined by preliminary dose–response experiments (0.1–10 μM) based on cell viability after irradiation. WR-2721 was used at 1.5 μM per established protocols.

#### 2.3.2. Long-Term Clonogenic Survival Assay

To evaluate the compound’s effect on long-term proliferative potential, a clonogenic assay was performed. Huh-7 cells were seeded at a low density (500 cells per well) in 6-well plates. After attachment, cells were pretreated with 0.15 μM TPP-C6-NIT for 1 h, followed by 6 Gy irradiation. After irradiation, the medium was replaced with complete medium, and cells were cultured for 10–14 days. Colonies were fixed with methanol, stained with 0.1% crystal violet, and those containing more than 50 cells were counted. The clonogenic survival rate was calculated as: (Number of colonies/Number of cells seeded) × 100%.

#### 2.3.3. Detection of Intracellular Reactive Oxygen Species (ROS) Levels

Intracellular total ROS levels were detected using the fluorescent probe DCFH-DA. Six hours post-irradiation, cells were collected and incubated with 10 μM DCFH-DA at 37 °C in the dark for 20 min. After washing three times with ice-cold PBS, fluorescence intensity was immediately analyzed using a flow cytometer (FACS, CytoFLEX Flow Cytometer, Beckman Coulter, Brea, CA, USA) at an excitation/emission wavelength of 488/525 nm or observed under a fluorescence microscope. At least 10,000 cells were analyzed per group, and the mean fluorescence intensity was used to quantify ROS levels.

#### 2.3.4. Measurement of Cellular Antioxidant Enzyme Activities and Lipid Peroxidation Product

Twenty-four hours post-irradiation, cells were collected, and cell lysates were prepared by sonication. Following the manufacturer’s instructions of the Nanjing Jiancheng Bioengineering Institute kits, the following were determined: Superoxide Dismutase (SOD) activity: Measured using the xanthine oxidase method. Results are expressed in units per milligram of protein (U/mg prot). Glutathione Peroxidase (GSH-Px) activity: Calculated by measuring the rate of GSH consumption. Results are expressed in units per milligram of protein (U/mg prot). Malondialdehyde (MDA) content: Determined using the thiobarbituric acid (TBA) method to assess lipid peroxidation levels. Results are expressed in nanomoles per milligram of protein (nmol/mg prot).

#### 2.3.5. Detection of Mitochondrial Membrane Potential (ΔΨ_m_)

Mitochondrial functional status was assessed using the fluorescent probe JC-1. Twelve hours post-irradiation, cells were collected and incubated with JC-1 working solution (5 μg/mL) at 37 °C in the dark for 20 min. After washing with PBS, dual-channel detection was performed using a flow cytometer: fluorescence was analyzed at emission wavelengths of 590 nm (red, aggregates, high ΔΨ_m_) and 530 nm (green, monomers, low ΔΨ_m_). Changes in mitochondrial membrane potential were quantified by the ratio of red-to-green fluorescence intensity, where a decrease in the ratio indicates depolarization.

#### 2.3.6. Measurement of Intracellular Iron Levels and GPX4 Activity

Intracellular Fe^2+^ and total iron levels were measured using commercial kits (Beijing Solarbio Science & Technology Co., Ltd., Beijing, China) according to the manufacturer’s instructions. Briefly, L-02 cells were collected 24 h post-irradiation, lysed, and incubated with FerroOrange fluorescent probe (for Fe^2+^) or total iron assay reagent. Fluorescence intensity (Ex/Em: 488/525 nm) was measured using a microplate reader. Fe^2+^/total iron ratio was calculated accordingly. GPX4 enzyme activity was measured using a GPX4 activity assay kit (Nanjing Jiancheng Bioengineering Institute, Nanjing, China) following the manufacturer’s protocol, based on NADPH oxidation measured at 340 nm.

### 2.4. Animal Experiments and In Vivo Radioprotection Evaluation

All animal experiments were approved by the Animal Care and Use Committee of the Air Force Medical University (AFMU) on 19 April 2023, following the Guidelines for the Care and Use of Laboratory Animals (Certificate No. 20230946). Male BALB/c mice were randomly divided into four groups (*n* = 10 per group): (1) Sham-irradiation control group; (2) Irradiation-only group (6 Gy whole-body irradiation); (3) TPP-C6-NIT treatment group (administered via tail vein injection); (4) WR-2721 positive control group. Survival rate and body weight changes were recorded daily after irradiation. On day 7 post-irradiation, blood and liver tissues were collected. Serum alanine aminotransferase (ALT) and aspartate aminotransferase (AST) activities were measured using commercial assay kits. Liver tissues were fixed in 10% formalin, followed by hematoxylin and eosin (H&E) staining to evaluate histopathological damage.

The in vivo dose of TPP-C6-NIT (10 mg/kg) was selected from a preliminary dose-finding study (5, 10, 20 mg/kg) as the optimal dose that provided consistent liver protection without observable toxicity.

### 2.5. Analysis of Tissue Oxidative Damage, Mitochondrial Function, and Related Protein Expression

#### 2.5.1. Detection of Hepatic Oxidative Stress Biomarkers

On day 7 post-irradiation, approximately 100 mg of fresh liver tissue was weighed, and a 10% liver tissue homogenate was prepared in ice-cold saline at a weight (g)-to-volume (mL) ratio of 1:9 using a homogenizer on ice. The homogenate was centrifuged at 4 °C, 3000× *g* for 10 min, and the supernatant was collected for subsequent assays. All assays were strictly performed following the kit instructions (Nanjing Jiancheng Bioengineering Institute): SOD activity: Measured using the xanthine oxidase method. One unit (U) of activity was defined as the amount of enzyme required to achieve 50% inhibition of the substrate oxidation reaction in 1 mL of reaction solution per mg of tissue protein. Results are expressed as U/mg prot. GSH-Px activity: Determined by measuring the consumption rate of GSH in the enzymatic reaction, using H_2_O_2_ as the substrate and monitoring NADPH consumption at 340 nm. Results are expressed as U/mg prot. MDA content: Detected using the thiobarbituric acid reactive substances (TBARS) method. Absorbance was measured at 532 nm, and the concentration was calculated based on a standard curve. Results are expressed as nmol/mg prot. GSH/GSSG ratio: The contents of reduced glutathione (GSH) and oxidized glutathione (GSSG) were determined separately using the DTNB colorimetric method and an enzymatic recycling assay, respectively. Their ratio was calculated to assess the overall tissue redox status.

#### 2.5.2. Evaluation of Hepatic Mitochondrial Function and Energy Metabolism

Tissue ATP content measurement: An ATP detection kit was used. Approximately 50 mg of liver tissue was lysed according to the manufacturer’s instructions, and ATP was quantified based on the bioluminescence intensity generated by the luciferase–luciferin system [15]. Results are expressed as nmol/mg prot. Mitochondrial Complex III (Ubiquinol: cytochrome c reductase) activity assay: Mitochondria were isolated from fresh liver tissue using a mitochondrial isolation kit. Enzyme activity was measured in a spectrophotometer by monitoring the rate of increase in absorbance at 550 nm due to the reduction in cytochrome c. Enzyme activity is expressed as nmol of reduced cytochrome c produced per minute per mg of mitochondrial protein (nmol/min/mg protein) and normalized to citrate synthase activity. In situ tissue ROS visualization: The dihydroethidium (DHE) fluorescent probe was used. Fresh liver tissues were embedded in OCT compound, and 8 μm thick frozen sections were prepared. The sections were incubated with 50 μM DHE working solution at 37 °C in the dark for 30 min, washed with PBS, and mounted with an anti-fade mounting medium containing DAPI. Sections were observed under a fluorescence microscope (excitation/emission: 518/605 nm), where red fluorescence intensity represents superoxide anion levels. The average fluorescence intensity of at least five random fields was semi-quantified using ImageJ (version 1.54p) software.

#### 2.5.3. Observation of Hepatic Mitochondrial Ultrastructure

On day 7 post-irradiation, mouse liver tissue was cut into approximately 1 mm^3^ pieces and immediately immersed in pre-cooled 2.5% glutaraldehyde (prepared in 0.1 M phosphate buffer, pH 7.4) for fixation at 4 °C for over 4 h. After thorough washing with 0.1 M phosphate buffer, the samples were post-fixed with 1% osmium tetroxide for 2 h. This was followed by dehydration through a graded acetone series, infiltration, and embedding in Epon812 resin. Ultrathin sections (60–80 nm) were prepared, double-stained with uranyl acetate and lead citrate, and observed under an HT7800 transmission electron microscope to capture images of hepatocyte mitochondrial morphology.

#### 2.5.4. Assessment of Mitochondrial Function (Seahorse Assay)

Real-time ATP production rate, mitochondrial stress, and glycolytic rate were measured using a Seahorse XFe24 Analyzer (Agilent Technologies, Santa Clara, CA, USA) [16]. L-02 cells were seeded into a Seahorse XFe24 V7 microplate (Agilent Technologies, Santa Clara, CA, USA) at a density of 5 × 10^3^ cells per well and divided into Control, X-ray, and TPP-C6-NIT (0.15 μM) + X-ray groups. The sensor cartridge in Seahorse XF Calibrant was hydrated overnight in a non-CO_2_ incubator at 37 °C. The culture medium was replaced with Agilent Seahorse XF DMEM medium supplemented with 1 mM sodium pyruvate, 2 mM glutamine, and 1 mM D-glucose. Cells were then incubated at 37 °C for 60 min prior to the assay, followed by washing with Seahorse buffer. Oligomycin (1.5 μM), FCCP (1.0 μM), and Rotenone/Antimycin A (Rot/AA, 0.5 μM) were automatically injected sequentially to measure the oxygen consumption rate (OCR). Protein concentration was determined using a BCA protein assay kit, and OCR values were normalized to total protein. All procedures were performed at 37 °C.

#### 2.5.5. Western Blot Analysis

Approximately 50 mg of liver tissue was homogenized on ice in RIPA lysis buffer containing protease and phosphatase inhibitors. The lysate was centrifuged at 4 °C, 12,000× *g* for 15 min, and the supernatant was collected as the total protein extract. Protein concentration was determined using the BCA method. Equal amounts of protein (typically 20–30 μg) were subjected to Western blotting analysis. Briefly, protein samples were separated by 10% SDS-PAGE and transferred onto a PVDF membrane using the wet transfer method. The membrane was blocked with 5% non-fat milk at room temperature for 1 h, followed by incubation with the corresponding primary antibodies at 4 °C overnight on a shaker. The primary antibodies used, focusing on redox homeostasis, and their dilutions were as follows: GPX4 (1:1000, GB154327), Nrf2 (1:1000, GB113808) and the loading control antibodies GAPDH (1:5000, GB15004) and β-actin (1:2000, GB12001). After primary antibody incubation, the membrane was thoroughly washed with TBST and incubated with the corresponding horseradish peroxidase (HRP)-conjugated secondary antibody at room temperature for 1 h. Finally, protein bands were visualized using a high-sensitivity ECL chemiluminescent substrate on a gel imaging system. The grayscale values of the protein bands were quantified using ImageJ software. The expression levels of target proteins were normalized to GAPDH and are presented and statistically analyzed as relative expression.

### 2.6. In Silico Molecular Docking Simulation

To preliminarily explore potential molecular interactions, molecular docking simulations were performed using the online platform CB-Dock2. The crystal structures of mitochondrial Complex III (PDB: 1BGY) and Keap1 (PDB: 3WN7) were retrieved from the Protein Data Bank. The three-dimensional structure of TPP-C6-NIT was obtained from the PubChem database or constructed and optimized using Chem3D. The docking results were analyzed and visualized based on binding energy and interaction modes.

### 2.7. Analysis of Apoptosis and Histopathology

#### 2.7.1. TUNEL Staining

A commercial TUNEL apoptosis detection kit was used. Paraffin-embedded sections were dewaxed, rehydrated, and treated with proteinase K, then incubated with the TUNEL reaction mixture at 37 °C in the dark for 1 h. After DAPI counterstaining, the sections were observed under a fluorescence microscope, where TUNEL-positive (green) signals indicated apoptotic cells.

#### 2.7.2. Liver Tissue H&E Staining

Fixed liver tissues were routinely embedded in paraffin, sectioned, and stained with hematoxylin and eosin (H&E). Morphological changes were observed under a light microscope.

### 2.8. Detection of Serum Liver Injury, Inflammatory, and Lipid Peroxidation Markers

#### 2.8.1. Liver Injury Markers

Commercial assay kits were used to measure serum ALT and AST activities following the manufacturer’s instructions. Results are expressed in U/L. Inflammatory cytokines: ELISA kits were used to measure the concentrations of IL-1β, TNF-α, and IL-6 in serum. Results are expressed in pg/mL.

#### 2.8.2. Lipid Peroxidation End Product

A 4-hydroxynonenal (4-HNE) ELISA kit was used to measure the content of 4-HNE in liver tissue homogenates. Results are expressed as nmol/mg prot. All assays were performed with technical replicates and independently repeated three times.

### 2.9. Liver Untargeted Metabolomics Analysis

To investigate the impact of radiation and TPP-C6-NIT intervention on the global metabolic profile of the liver, particularly the lipid metabolism network, untargeted metabolomics analysis was performed on liver tissue. Briefly, approximately 30 mg of liver tissue was used to extract metabolites with pre-cooled 80% methanol aqueous solution. Chromatographic separation and mass spectrometry data acquisition were performed using a UHPLC-Q Exactive HF-X system (Thermo Fisher Scientific, Waltham, MA, USA). After preprocessing the raw data, multivariate statistical analysis (PCA and OPLS-DA) was conducted to screen differential metabolites with VIP > 1.0 and *p* < 0.05. The differential metabolites were primarily focused on lipids and related metabolites and were functionally annotated using the KEGG and LIPIDMAPS databases, aiming to reveal metabolic reprogramming patterns associated with oxidative damage.

### 2.10. Statistical Analysis

All data are presented as the mean ± standard deviation (SD) and were processed using GraphPad Prism 8.0 software. Comparisons between two groups were performed using an unpaired Student’s *t*-test. Comparisons among multiple groups were performed using one-way analysis of variance (ANOVA), followed by Tukey’s test for post hoc multiple comparisons. Survival analysis was performed using the Log-rank test. A *p*-value < 0.05 was considered statistically significant.

## 3. Results

### 3.1. Design, Synthesis, and In Vitro Antioxidant Activity Characterization of TPP-C6-NIT

**Figure 1:**
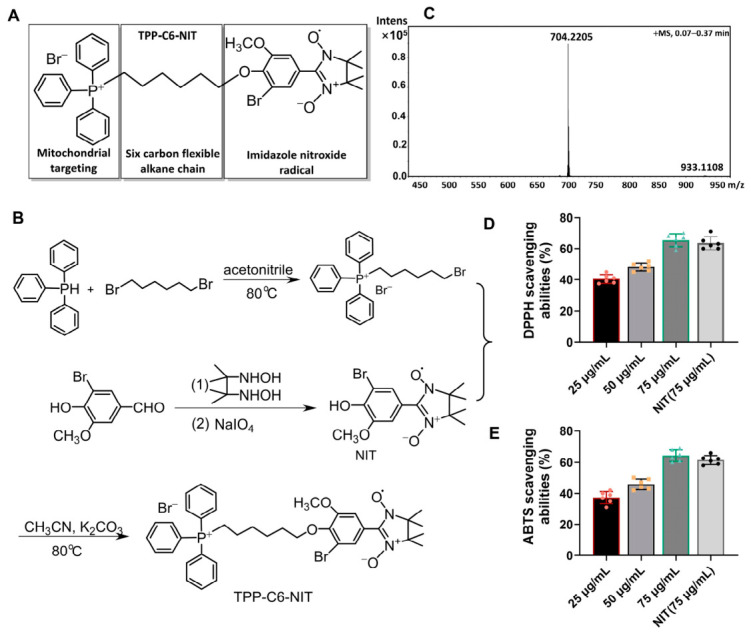
Chemical design, synthesis, and in vitro antioxidant activity of Br-TPP-NIT. (**A**) Schematic illustration of the chemical structure of TPP-C6-NIT. The molecule consists of three components: a triphenylphosphonium (TPP^+^) targeting moiety, a six-carbon (C6) linker chain, and an imidazole nitroxide radical (NIT) antioxidant moiety. (**B**) Simplified scheme of the key synthetic route for TPP-C6-NIT. (**C**) High-resolution mass spectrometry (HRMS) spectrum of TPP-C6-NIT, showing the molecular ion peak [M-Br]^+^. (**D**,**E**) In vitro DPPH radical scavenging activity (**D**) and ABTS radical scavenging activity (**E**) of TPP-C6-NIT and its non-targeting analog, NIT. Data are presented as the mean ± standard deviation (SD) (*n* = 6).

To construct an antioxidant capable of targeting the core site of radiation damage, we designed and synthesized a novel molecule designed to target mitochondria, TPP-C6-NIT. Its design was based on a “targeting moiety–linker–effector moiety” strategy (Figure 1A): a triphenylphosphonium cation serves as the mitochondrial-targeting head, covalently linked via a flexible six-carbon alkane chain to a free radical-scavenging imidazole nitroxide radical. This architecture is intended to utilize the dependence of TPP^+^ on the mitochondrial membrane potential (ΔΨ_m_), with the aim of guiding the molecule to preferentially accumulate within the mitochondrial matrix.

We successfully synthesized TPP-C6-NIT through a concise route (synthetic scheme shown in Figure 1B). The chemical structure was confirmed by high-resolution mass spectrometry (HRMS). The observed molecular ion peak [M]^+^ was in full agreement with the theoretical value (Figure 1C), confirming the successful synthesis of the target compound.

The in vitro antioxidant capacity of TPP-C6-NIT was evaluated using DPPH and ABTS radical scavenging assays. The results showed that TPP-C6-NIT exhibited dose-dependent scavenging activity against both radicals (Figure 1D,E). At an equivalent concentration (75 μg/mL), its scavenging rate was comparable to that of the NIT moiety without the conjugated TPP^+^, indicating that linking the TPP^+^ unit did not compromise the intrinsic antioxidant activity of the imidazole nitroxide radical. Together, these results indicate that TPP-C6-NIT is a novel compound with both mitochondrial-targeting potential and potent antioxidant capacity in vitro.

### 3.2. Protective Effects of TPP-C6-NIT on Normal Hepatocytes Post-Irradiation: Mitigating Oxidative Stress

**Figure 2:**
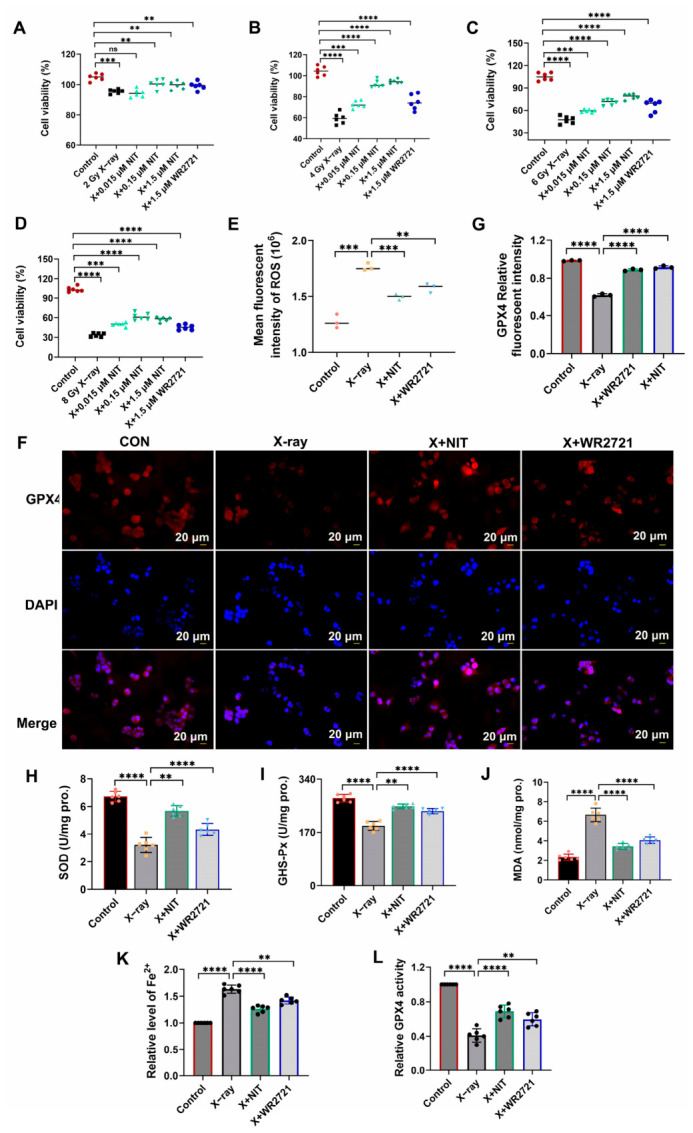
TPP-C6-NIT protects normal hepatocytes from radiation-induced oxidative damage. (**A**–**D**) Cell viability was determined 72 h after irradiation. Cells were pretreated with different concentrations of TPP-C6-NIT or WR-2721 before receiving X-ray irradiation at various doses (0–8 Gy) (*n* = 6). (**E**) Intracellular ROS levels 6 h post-irradiation were quantitatively analyzed by flow cytometry. Data are expressed as relative fluorescence intensity (*n* = 3). (**F**) Representative immunofluorescence staining images of GPX4 protein (scale bar = 20 μm). (**G**) Quantitative analysis of the mean fluorescence intensity (*n* = 3). (**H**–**J**) Levels of antioxidant enzyme activities (SOD, GSH-Px) and lipid peroxidation product (MDA) in cells 24 h post-irradiation (*n* = 6). (**K**) Intracellular Fe^2+^ levels measured 24 h post-irradiation, normalized to the control group (*n* = 6). (**L**) GPX4 enzyme activity measured 24 h post-irradiation, normalized to the control group (*n* = 6). Data are presented as the mean ± SD. ** *p* < 0.01, *** *p* < 0.001, **** *p* < 0.0001, ns, not significant.

After confirming the in vitro antioxidant activity of TPP-C6-NIT, we further evaluated its protective effects against radiation damage at the cellular level. First, its impact on the viability of normal human hepatocytes L-02 was assessed using the CCK-8 assay. As shown in Figure 2A–D, the survival rate of L-02 cells exhibited a dose-dependent decrease following X-ray irradiation (2–8 Gy). However, after a 1 h pretreatment with TPP-C6-NIT (0.15–0.6 μM), cell viability was significantly enhanced. At 6 Gy irradiation, 0.6 μM TPP-C6-NIT increased the cell survival rate from approximately 45% in the irradiation-only group to over 80%, an effect superior or comparable to that of the positive control WR-2721 (1.5 μM). These results indicate that TPP-C6-NIT effectively maintains the viability of hepatocytes after irradiation.

Radiation-induced overproduction of free radicals is a key mediator of cellular damage. Using flow cytometry, we found that TPP-C6-NIT pretreatment significantly reduced radiation-induced ROS production in L-02 cells (Figure 2E).

We also examined GPX4 expression by immunofluorescence staining (Figure 2F,G). In the normal control group, cells displayed high-intensity GPX4 fluorescence signals (red), uniformly distributed in the cytoplasm, with intact morphology and clear nuclear staining (DAPI, blue). In the irradiation group, the GPX4 fluorescence signal was markedly attenuated, with some cells showing fragmented or punctate fluorescence; concurrently, cell shrinkage and nuclear pyknosis were observed. In cells pretreated with TPP-C6-NIT, GPX4 fluorescence intensity was partially restored, and cell morphology was improved. These observations indicate that TPP-C6-NIT treatment is associated with preserved GPX4 expression under radiation exposure.

To assess the impact on the cellular antioxidant defense system, we measured the activities of key antioxidant enzymes and the level of lipid peroxidation. The results showed that radiation significantly reduced intracellular SOD and GSH-Px activities while increasing the content of malondialdehyde (MDA), a lipid peroxidation end product. Pretreatment with TPP-C6-NIT significantly reversed these changes, restoring SOD and GSH-Px activities and reducing MDA levels (Figure 2H–J).

To further characterize the oxidative stress profile in L-02 cells, we measured intracellular Fe^2+^ levels and GPX4 enzyme activity. As shown in Figure 2K, radiation significantly increased intracellular Fe^2+^ levels, which were reduced by TPP-C6-NIT or WR-2721 pretreatment. Additionally, radiation markedly decreased GPX4 activity, and this effect was significantly reversed by TPP-C6-NIT or WR-2721 treatment (Figure 2L). These data provide additional evidence that TPP-C6-NIT alleviates radiation-induced oxidative stress and preserves antioxidant capacity in normal hepatocytes.

In summary, at the cellular level, TPP-C6-NIT reduces radiation-induced oxidative stress in normal hepatocytes and is associated with enhanced endogenous antioxidant capacity.

### 3.3. Molecular Docking Exploration of Potential Targets

To gain preliminary insight into possible molecular interactions underlying the effects of TPP-C6-NIT, we performed molecular docking studies.

**Figure 3:**
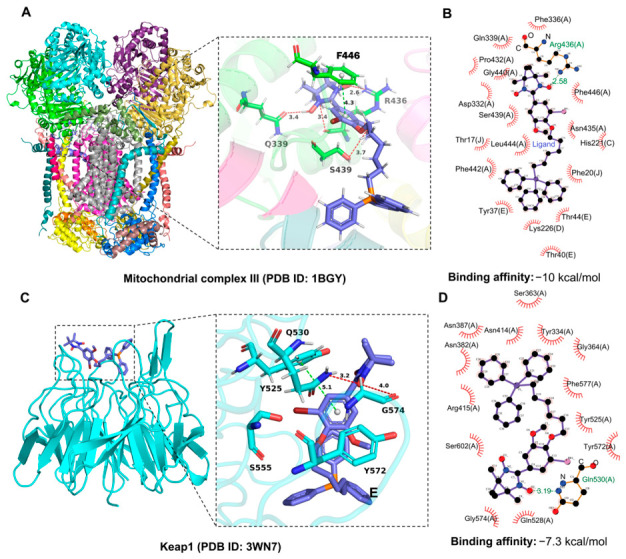
Molecular docking of TPP-C6-NIT with potential targets (exploratory). (**A**) Molecular docking pose of TPP-C6-NIT within mitochondrial Complex III (PDB: 1BGY). Key interacting residues are labeled. (**B**) Two-dimensional interaction diagram showing the detailed binding interactions (hydrogen bonds and π–π stacking) between TPP-C6-NIT and mitochondrial Complex III. (**C**) Molecular docking model of TPP-C6-NIT (purple sticks) bound to the Kelch domain of Keap1 (PDB: 3WN7). TPP-C6-NIT precisely occupies the natural Nrf2-binding pocket, with its benzene ring forming a π–π stack (green dashed lines) with Tyr525, and the oxygen atom on the imidazole ring forming hydrogen bonds (red dashed lines) with Gln530 and Gly574. (**D**) Two-dimensional interaction diagram showing the binding interactions between the native Nrf2 peptide and the Kelch domain of Keap1 (PDB: 3WN7), shown as a reference for comparison with (**C**).

Given its mitochondria-targeting design, we first examined its potential interaction with mitochondrial Complex III (ubiquinol–cytochrome c reductase), a key component of the electron transport chain. The docking results predicted that TPP-C6-NIT could bind to the hydrophobic pocket of Complex III (Figure 3A), with key interactions summarized in a two-dimensional diagram (Figure 3B). Additionally, given the observed changes in Nrf2 expression upon TPP-C6-NIT treatment, we also examined its potential interaction with Keap1 (PDB: 3WN7), the key negative regulator of Nrf2. The results showed that TPP-C6-NIT was predicted to bind to the Kelch domain of Keap1, occupying the natural Nrf2-binding pocket (Figure 3C). Detailed analysis suggested that the benzene ring of TPP-C6-NIT could form a π–π stacking interaction with Tyr525, and the oxygen atom on the imidazole ring could form hydrogen bonds with Gln530 and Gly574 (Figure 3C). The binding conformation of the native Nrf2 peptide is shown as a reference (Figure 3D).

These computational predictions are exploratory and do not constitute direct evidence of target engagement. Experimental validation is required to confirm these potential interactions and their biological relevance.

### 3.4. TPP-C6-NIT Exhibits Minimal Protective Effect in Hepatocellular Carcinoma Cells, Suggesting Possible Selective Cytoprotection

An ideal radioprotective agent should safeguard normal tissues without compromising the therapeutic efficacy of radiotherapy against tumor cells. To evaluate whether TPP-C6-NIT exhibits such selectivity, we conducted parallel experiments in human hepatocellular carcinoma Huh-7 cells.

**Figure 4:**
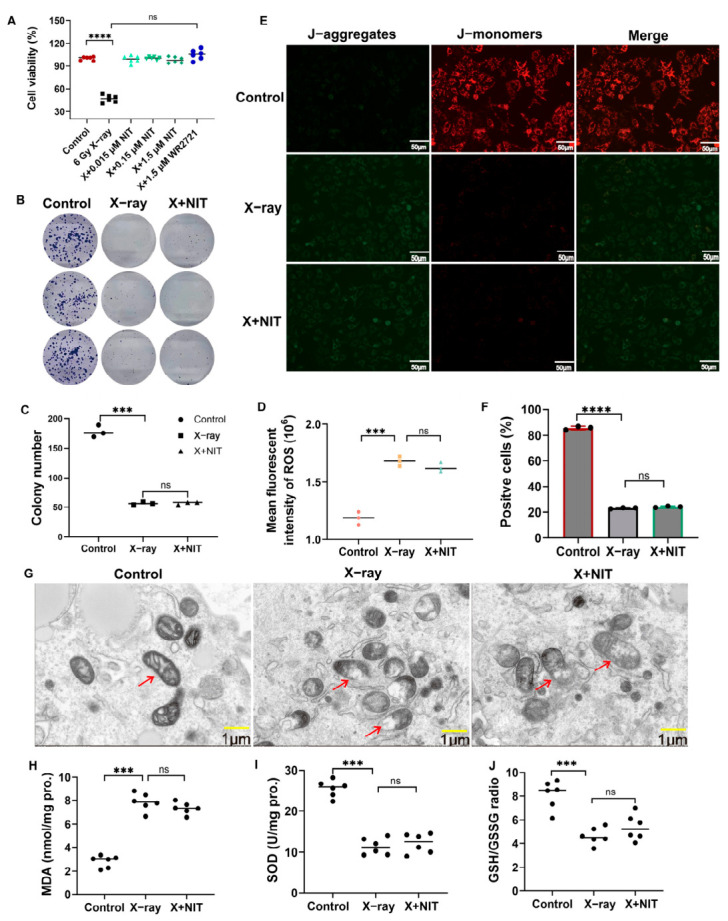
TPP-C6-NIT exhibits minimal protective effect in hepatocellular carcinoma cells. (**A**) Effect of pretreatment with TPP-C6-NIT (0.15 μM) on the viability of Huh-7 cells 72 h after 6 Gy irradiation, as determined by the CCK-8 assay (*n* = 6). (**B**) Long-term proliferative potential of Huh-7 cells assessed by the clonogenic survival assay. (**C**) Quantitative analysis of the clonogenic survival rate (*n* = 3). (**D**) Intracellular ROS levels in Huh-7 cells post-irradiation were quantitatively analyzed by flow cytometry. Pretreatment with TPP-C6-NIT failed to significantly reduce ROS (*n* = 3). (**E**) Mitochondrial membrane potential (ΔΨ_m_) assessed by the JC-1 fluorescent probe. (**F**) Quantitative analysis of the JC-1 red/green fluorescence intensity ratio in Huh-7 cells (*n* = 3). (**G**) Transmission electron microscopy images of mitochondrial ultrastructure of Huh-7 cells (scale bar = 1 μm). Arrows indicate normal mitochondrial cristae structure; swelling and cristae rupture in mitochondria after irradiation. (**H**) MDA content of Huh-7 cells (*n* = 6). (**I**) SOD activity of Huh-7 cells (*n* = 6). (**J**) GSH/GSSG ratio of Huh-7 cells (*n* = 6). All quantitative data are presented as the mean ± SD. *** *p* < 0.001, **** *p* < 0.0001, ns, not significant.

First, we tested the effect of TPP-C6-NIT on cell viability across various radiation doses (2–8 Gy). As shown in Figure 4A, at 6 Gy irradiation, the survival rate of Huh-7 cells 72 h after pretreatment with TPP-C6-NIT (0.15 μM) showed no statistically significant difference compared to the irradiation-only group. The long-term clonogenic survival assay further confirmed that TPP-C6-NIT did not improve the proliferative potential of Huh-7 cells after irradiation (Figure 4B,C).

We further investigated the impact of TPP-C6-NIT on oxidative stress and mitochondrial function in Huh-7 cells. In contrast to its ROS-scavenging effect in normal L-02 hepatocytes, pretreatment with TPP-C6-NIT failed to significantly reduce radiation-induced intracellular ROS levels in Huh-7 cells (Figure 4D). JC-1 staining revealed that TPP-C6-NIT did not stabilize the mitochondrial membrane potential (ΔΨ_m_) in irradiated Huh-7 cells, as the decrease in the red/green fluorescence intensity ratio was comparable to that in the irradiation-only group (Figure 4E,F).

Transmission electron microscopy further showed that TPP-C6-NIT pretreatment did not prevent radiation-induced mitochondrial ultrastructural damage in Huh-7 cells, including marked swelling and cristae rupture (Figure 4G). Consistently, TPP-C6-NIT failed to reverse the radiation-induced elevation of malondialdehyde (MDA) (Figure 4H), the reduction in superoxide dismutase (SOD) activity (Figure 4I), or the disturbance of the GSH/GSSG ratio (Figure 4J).

Thus, under identical experimental conditions, TPP-C6-NIT showed comprehensive protection in normal L-02 hepatocytes but no significant protection in Huh-7 hepatocellular carcinoma cells. This lack of protection in Huh-7 cells is an interesting finding that suggests possible selective cytoprotection. The underlying mechanisms remain to be determined but may involve fundamental differences in redox homeostasis or mitochondrial function between normal and cancer cells.

### 3.5. TPP-C6-NIT Improves Survival and Alleviates Liver Injury in Whole-Body Irradiated Mice

After confirming the protective effects of TPP-C6-NIT at the cellular level, we further evaluated its efficacy in a mouse model of whole-body irradiation, with a focus on liver protection.

**Figure 5:**
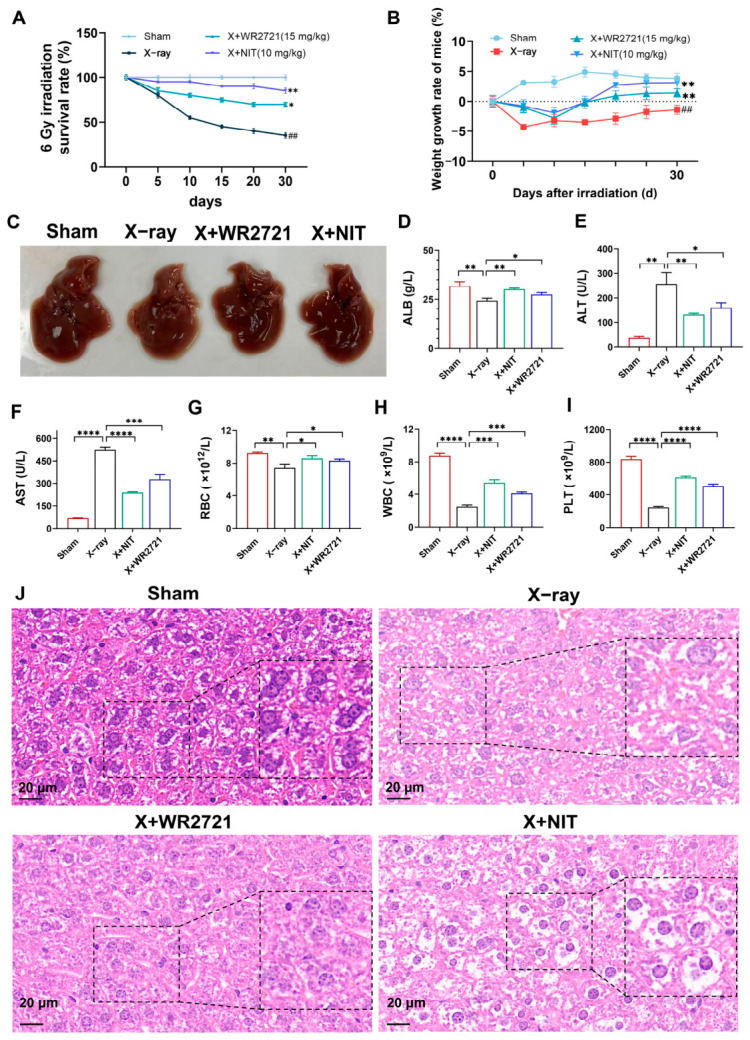
In vivo protective effects of TPP-C6-NIT on whole-body irradiated mice. (**A**) Thirty-day survival curves of mice (*n* = 10). TPP-C6-NIT (10 mg/kg) or WR-2721 (15 mg/kg) was administered via tail vein injection 1 h before irradiation. (**B**) Changes in mouse body weight after irradiation (normalized to body weight on day 0) (*n* = 10). (**C**) Representative photographs of gross liver morphology from each group on day 7 post-irradiation. (**D**–**F**) Serum ALB, ALT and AST activities in mice on day 7 post-irradiation (*n* = 6). (**G**–**I**) Peripheral red blood cell (RBC), white blood cell (WBC), and platelet (PLT) counts in mice on day 7 post-irradiation (*n* = 6). (**J**) Representative images of liver tissue in mice stained with hematoxylin and eosin (H&E) (scale bar = 20 μm). The dashed box indicates the area shown at higher magnification in the adjacent image. All data are presented as the mean ± SD. * *p* < 0.05, ** *p* < 0.01, *** *p* < 0.001, **** *p* < 0.0001 vs. sham group; ## *p* < 0.01 vs. X-ray group.

A single 6 Gy whole-body irradiation resulted in 100% mortality within 30 days, with a median survival of only 14 days. In contrast, pretreatment with TPP-C6-NIT (10 mg/kg) significantly extended the median survival to 28 days and increased the 30-day survival rate to 60%, an effect comparable to or better than that of the positive control WR-2721 (15 mg/kg) (Figure 5A). Concurrently, TPP-C6-NIT mitigated the progressive weight loss induced by irradiation (Figure 5B). Gross liver morphology showed that after irradiation, the liver appeared dull and reduced in size, whereas the liver in the TPP-C6-NIT pretreatment group appeared closer to that of the sham-irradiated group, being ruddy and plump (Figure 5C).

On day 7 post-irradiation, serum ALB, ALT and AST activities were sharply elevated in the irradiation-only group, indicating hepatocellular damage. Pretreatment with TPP-C6-NIT significantly suppressed the increase in both transaminases, reducing their levels by approximately 55% (Figure 5D–F). Furthermore, the irradiation-induced suppression of peripheral red blood cell, white blood cell, and platelet counts was also ameliorated by TPP-C6-NIT (Figure 5G–I), suggesting a potential protective effect on the hematopoietic system.

H&E staining showed clear hepatic lobule structures and orderly arranged hepatocytes in the sham group. In contrast, the irradiation-only group exhibited diffuse damage, including disordered hepatic cord structures, significant hepatocyte edema with ballooning degeneration, and scattered punctate necrotic foci. TPP-C6-NIT pretreatment significantly alleviated these pathological alterations (Figure 5J).

Thus, at the whole-animal level, TPP-C6-NIT improved survival and body weight maintenance, protected liver function, and alleviated histological damage. These results indicate that TPP-C6-NIT provides protection against radiation-induced liver injury in vivo.

### 3.6. TPP-C6-NIT Protects Mitochondrial Structure and Function

Based on the mitochondria-targeting design of TPP-C6-NIT, we further evaluated its effects on mitochondria after irradiation.

**Figure 6:**
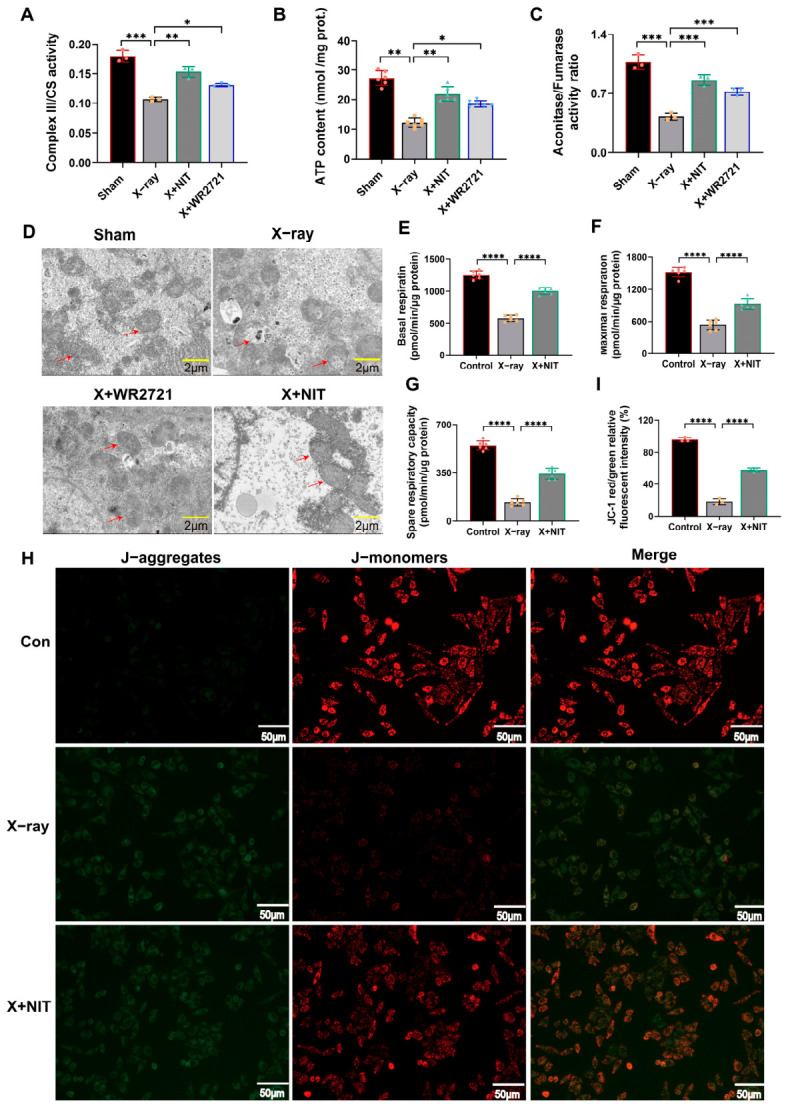
TPP-C6-NIT protects mitochondrial function and structure. (**A**) Maximal enzymatic activity of mitochondrial Complex III in liver tissue, normalized to citrate synthase (CS) activity (*n* = 3). (**B**) ATP content in liver tissue determined using a commercial kit (*n* = 6). (**C**) The aconitase-to-fumarase activity ratio, where fumarase serves as a specific biomarker for mitochondrial oxidative stress (*n* = 6). (**D**) Representative transmission electron microscopy (TEM) images of hepatocytes in liver tissue (scale bar = 2 μm). Arrows indicate normal mitochondrial cristae structure, swelling, cristae rupture, and vacuolization in mitochondria after irradiation; and relatively intact mitochondrial morphology under the protection of TPP-C6-NIT. (**E**–**G**) TPP-C6-NIT alleviates X-ray radiation-induced mitochondrial respiratory dysfunction in L-02 cells: (**E**) basal respiration, (**F**) maximal respiration, and (**G**) spare respiratory capacity. (**H**) JC-1 flow cytometry scatter plot (lower right quadrant: red fluorescence, high ΔΨ_m_; upper right quadrant: dual red/green positive, loss of ΔΨ_m_). (**I**) Quantitative analysis of the red/green fluorescence intensity ratio (*n* = 3). All data are presented as the mean ± SD. * *p* < 0.05, ** *p* < 0.01, *** *p* < 0.001, **** *p* < 0.0001.

At the whole-animal level, we assessed liver mitochondrial energy metabolism. On day 7 post-irradiation, the maximal enzymatic activity of mitochondrial Complex III in mouse liver was significantly decreased, and hepatic ATP content was also drastically reduced (Figure 6A,B). The ratio of aconitase to fumarase activity, a biomarker of mitochondrial oxidative stress, was also significantly reduced after irradiation and restored by TPP-C6-NIT pretreatment (Figure 6C). These results indicate that radiation impaired mitochondrial electron transport chain function and induced mitochondrial oxidative stress, both of which were alleviated by TPP-C6-NIT.

Transmission electron microscopy (TEM) analysis was performed to directly visualize mitochondrial ultrastructure. In hepatocytes from the sham-irradiated group, mitochondria exhibited typical oval or rod shapes with clear and tightly packed cristae (Figure 6D). In the irradiation-only group, mitochondria showed severe damage: most were markedly swollen, with fractured or absent cristae and vacuolized matrix. In the TPP-C6-NIT pretreatment group, mitochondrial morphology was substantially preserved, with reduced swelling and partial retention of cristae structure (Figure 6D).

We also measured the oxygen consumption rate (OCR) of L-02 cells using a Seahorse XFe24 Analyzer. TPP-C6-NIT pretreatment restored basal respiration (by 82%), maximal respiration, and spare respiratory capacity (by approximately 62%) (Figure 6E–G).

At the cellular level, we assessed mitochondrial membrane potential (ΔΨ_m_) using the JC-1 probe. In normal L-02 cells, JC-1 aggregates within mitochondria, emitting strong red fluorescence (high ΔΨ_m_). After 6 Gy irradiation, red fluorescence sharply decreased while green fluorescence (monomer, low ΔΨ_m_) increased, leading to a significant decline in the red/green fluorescence ratio (Figure 6H,I), indicating depolarization of ΔΨ_m_. Pretreatment with TPP-C6-NIT significantly stabilized ΔΨ_m_, resulting in recovery of the red/green fluorescence ratio (Figure 6I).

In summary, TPP-C6-NIT preserved mitochondrial energy metabolism (Complex III activity, ATP, aconitase/fumarase ratio) and ultrastructure in vivo, and maintained mitochondrial membrane potential and respiratory function in vitro. These findings suggest that mitochondrial protection is a key feature of TPP-C6-NIT’s radioprotective effects. 

### 3.7. TPP-C6-NIT Is Associated with Changes in Nrf2 and GPX4 Expression and Reduced Oxidative Stress in the Liver

To further explore the molecular features associated with TPP-C6-NIT treatment, we examined the expression of Nrf2 and GPX4 in the livers of irradiated mice.

**Figure 7:**
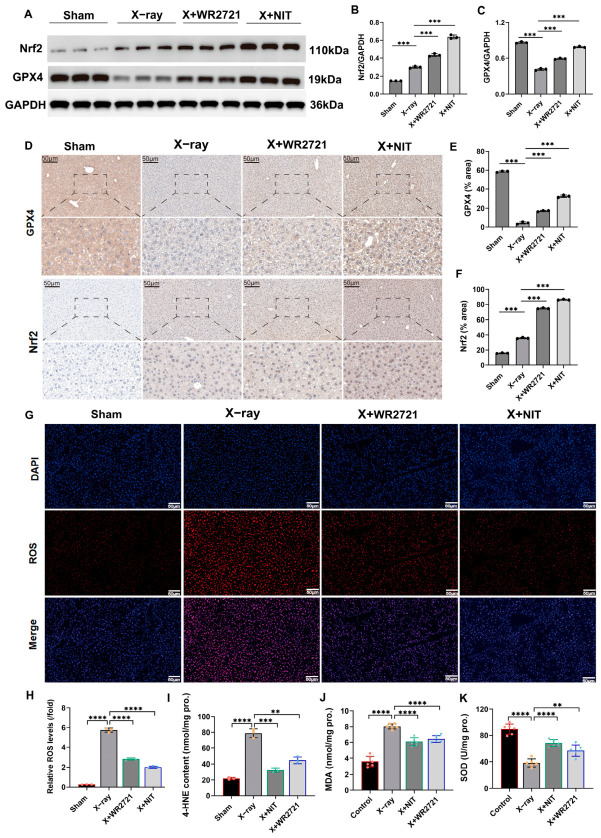
TPP-C6-NIT is associated with changes in Nrf2/GPX4 expression and reduced oxidative stress in the liver. (**A**–**C**) Western blot analysis and quantification of Nrf2 and GPX4 proteins in liver tissue (*n* = 3). (**D**–**F**) Immunohistochemical staining of Nrf2 and GPX4 in liver tissue (scale bar: 50 μm) and quantitative analysis (*n* = 3). (**G**,**H**) In situ ROS detection in liver tissue (red: ROS, blue: DAPI, scale bar: 50 μm) and quantitative analysis. (**I**) 4-HNE content in liver tissue (*n* = 3). (**J**) MDA content in liver tissue (*n* = 6). (**K**) SOD activity in liver tissue (*n* = 6). All data are presented as the mean ± SD. ** *p* < 0.01, *** *p* < 0.001, **** *p* < 0.0001.

Western blot analysis showed that compared to the sham-irradiated group, Nrf2 protein expression was increased in the irradiation-only group, while GPX4 expression was significantly decreased. In the TPP-C6-NIT pretreatment group, Nrf2 expression was further increased, and the reduction in GPX4 was reversed (Figure 7A–C).

Immunohistochemical staining confirmed these observations at the tissue level. Nrf2-positive signals in hepatocytes were enhanced after irradiation, and TPP-C6-NIT treatment further increased this signal. Conversely, the diminished GPX4 signal after irradiation was restored by TPP-C6-NIT pretreatment (Figure 7D–F).

Consistent with these observations, in situ ROS detection showed that TPP-C6-NIT treatment significantly reduced radiation-induced ROS production in liver tissue (Figure 7G,H). Quantitative analysis of the lipid peroxidation end product 4-hydroxynonenal (4-HNE) further confirmed that TPP-C6-NIT reversed the radiation-induced accumulation of 4-HNE (Figure 7I). Additionally, TPP-C6-NIT pretreatment reduced MDA levels and restored SOD activity in liver tissue (Figure 7J,K).

In summary, TPP-C6-NIT treatment was associated with increased Nrf2 expression, restored GPX4 expression, reduced oxidative stress markers (ROS, 4-HNE, MDA), and restored SOD activity in irradiated mouse liver. These observations suggest a link between TPP-C6-NIT and enhanced antioxidant capacity, although the underlying molecular mechanisms require further investigation.

### 3.8. Metabolomics Reveals That TPP-C6-NIT Is Associated with Reversal of Radiation-Induced Hepatic Lipid Metabolic Changes

To explore metabolic changes associated with TPP-C6-NIT treatment, we performed untargeted metabolomics analysis on mouse liver.

**Figure 8:**
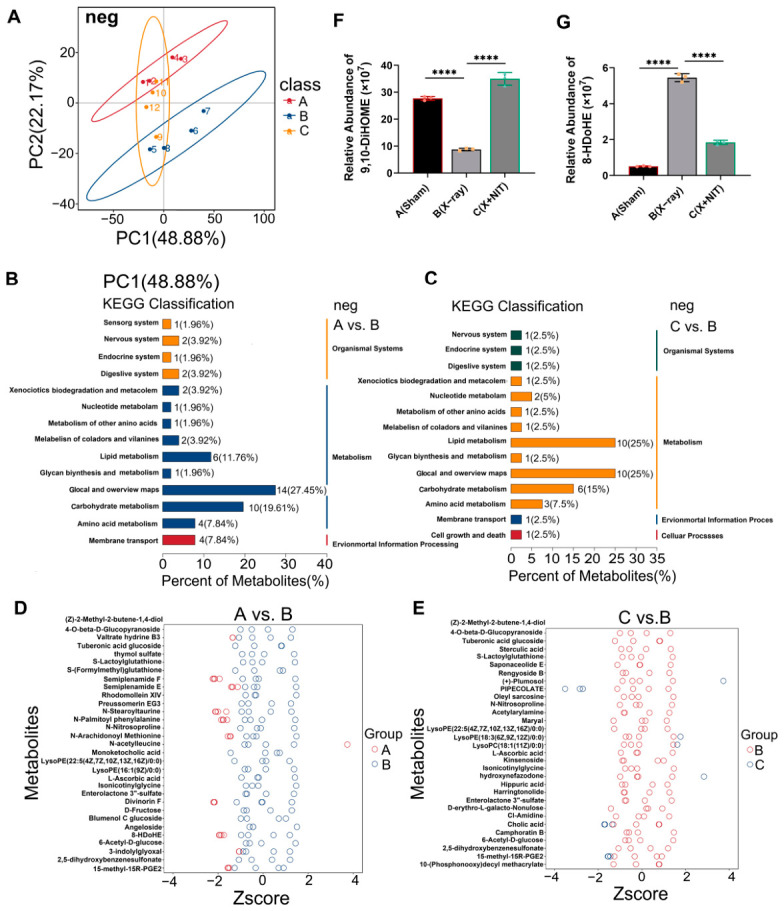
TPP-C6-NIT reverses radiation-induced hepatic metabolomic disorders. (**A**) Principal component analysis (PCA) score plot of liver metabolites based on negative ion mode data. The first two principal components (PC1 and PC2) explained 48.88% and 22.17% of the total variance, respectively. The plot shows the metabolic profile distribution of the sham-irradiated (Sham), irradiation-only (X-ray), and TPP-C6-NIT treatment (X-ray + TPP-C6-NIT) groups, with the trajectory of the treatment group reverting towards the sham group. (**B**,**C**) KEGG pathway enrichment analysis bubble charts: (**B**) irradiation-only group vs. sham-irradiated group (A vs. B), showing pathways significantly perturbed by radiation, such as linoleic acid metabolism; (**C**) TPP-C6-NIT treatment group vs. irradiation-only group (C vs. B), showing pathways significantly reversed by the compound, with linoleic acid metabolism being one of the most prominent. (**D**,**E**) Z-score clustered heatmaps of differential metabolites. Colors represent the relative abundance after Z-score normalization (red: high expression; blue: low expression). (**D**) Comparison of group A vs. B, showing widespread metabolic perturbations induced by radiation. (**E**) Comparison of group C vs. B, showing the specific reversal by TPP-C6-NIT of aberrant expression for a subset of metabolites. (**F**,**G**) Quantitative analysis of key metabolites related to oxidative stress: (**F**) relative abundance of the linoleic acid metabolism intermediate 9,10-DiHOME across the three groups; (**G**) relative abundance of the lipid peroxidation marker 8-HDoHE across the three groups. Data are presented as the mean ± SD. **** *p* < 0.0001.

Principal component analysis (PCA) revealed distinct separation of the metabolic profiles among the sham-irradiated, irradiation-only, and TPP-C6-NIT treatment groups. Notably, the metabolic profile of the treatment group was positioned between the irradiation-only group and the sham-irradiated group, showing a trend toward the sham group (Figure 8A).

Comparative analysis showed that radiation induced extensive metabolic changes. KEGG pathway enrichment analysis indicated that lipid-related pathways, such as linoleic acid metabolism and unsaturated fatty acid biosynthesis, were significantly perturbed (Figure 8B). Direct comparison between the treatment group and the irradiation-only group showed that the linoleic acid metabolism pathway was among those most notably associated with TPP-C6-NIT treatment (Figure 8C). Quantitative analysis of key metabolites in this pathway confirmed that TPP-C6-NIT treatment was associated with restored levels of metabolites that were altered in the irradiation group, such as 9,10-DiHOME, an intermediate of linoleic acid metabolism (Figure 8F). Concurrently, the elevated level of the lipid peroxidation marker 8-HDoHE after irradiation was also lower in the TPP-C6-NIT treatment group (Figure 8G). The cluster heatmap of differential metabolites further illustrated that TPP-C6-NIT treatment was associated with reversed expression of a subset of radiation-induced metabolites (Figure 8D,E).

Further analysis showed that N-acyl amino acid compounds derived from arachidonic acid (e.g., N-Arachidonoyl Methionine), which accumulated after radiation, were also lower in the TPP-C6-NIT treatment group. These compounds are conjugated products resulting from the oxidative degradation of polyunsaturated fatty acids. Their reduced levels following TPP-C6-NIT treatment are consistent with reduced lipid peroxidation. The detailed metabolomics data, including PCA, OPLS-DA, volcano plots, and KEGG pathway enrichment analyses, are provided in the Supplementary Materials (Figures S1–S6).

### 3.9. TPP-C6-NIT Reduces Radiation-Induced Apoptosis and Systemic Inflammation in the Liver

We further evaluated the effects of TPP-C6-NIT on radiation-induced apoptosis and systemic inflammation.

**Figure 9:**
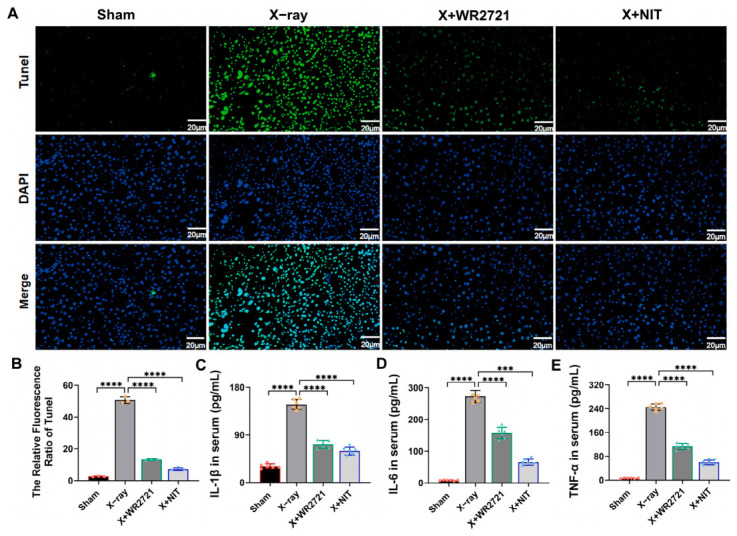
TPP-C6-NIT reduces radiation-induced apoptosis and systemic inflammation. (**A**) Representative TUNEL staining images of mouse liver tissue (green: apoptotic cells, blue: DAPI, scale bar: 20 μm). (**B**) Quantitative analysis of TUNEL-positive cells (*n* = 3). (**C**–**E**) Serum levels of pro-inflammatory cytokines IL-1β, IL-6, and TNF-α measured by ELISA (*n* = 6). All data are presented as the mean ± SD. *** *p* < 0.001, **** *p* < 0.0001.

TUNEL staining of mouse liver tissue showed that radiation induced a large number of TUNEL-positive apoptotic cells, while TPP-C6-NIT treatment markedly reduced the positive signal (Figure 9A,B). ELISA assays revealed that the levels of pro-inflammatory cytokines IL-1β, IL-6, and TNF-α in mouse serum were significantly elevated after radiation. TPP-C6-NIT treatment significantly reduced the levels of these inflammatory factors (Figure 9C–E). TPP-C6-NIT treatment was associated with reduced apoptosis in liver tissue and reduced systemic inflammation after irradiation.

## 4. Discussion

Radiation-induced liver injury (RILI) is a major complication limiting the efficacy of abdominal tumor radiotherapy, with mitochondrial oxidative stress as a central pathological event [4,5,17,18]. In this study, we designed and synthesized a mitochondria-targeted antioxidant, TPP-C6-NIT, and evaluated its protective effects in a whole-body irradiation mouse model with a focus on radiation-induced liver injury [19,20].

Radiation-generated ROS primarily originates from the dysfunction of the mitochondrial electron transport chain [21,22]. Our research indicates that TPP-C6-NIT exhibited potent free radical scavenging activity in vitro. In irradiated L-02 hepatocytes, it reduced ROS production, preserved mitochondrial membrane potential and respiratory function, and improved cell viability. In a mouse model of whole-body irradiation, TPP-C6-NIT pretreatment improved survival, reduced serum ALT/AST levels, alleviated liver histopathological damage, and reduced apoptosis and systemic inflammation. Notably, TPP-C6-NIT showed minimal protective effects in Huh-7 hepatocellular carcinoma cells, indicating a degree of selective cytoprotection.

Given its TPP^+^ moiety, TPP-C6-NIT was designed to target mitochondria. Our results showed that TPP-C6-NIT preserved mitochondrial Complex III activity and ATP levels in vivo, maintained mitochondrial membrane potential and respiratory function in vitro, and reduced radiation-induced mitochondrial swelling and cristae rupture at the ultrastructural level (Figure 6). This indicates that it not only protects the structure but also rapidly repairs the immediate function and metabolic flexibility of the oxidative phosphorylation system, thereby securing the energy supply required for cells to cope with radiation stress. This targeted maintenance of the “cellular power plants” constitutes the central link in its protective action [23,24,25].

We observed that TPP-C6-NIT treatment was associated with increased Nrf2 expression and restored GPX4 expression in irradiated mouse liver (Figure 7). Nrf2 acts as the “master regulator” of cellular antioxidant responses, while GPX4 is a crucial enzyme responsible for repairing lipid peroxidation and maintaining membrane structural stability [26,27,28,29,30]. Linoleic acid, as a polyunsaturated fatty acid, is a primary substrate for lipid peroxidation [31,32]. Metabolomics analysis further revealed that TPP-C6-NIT treatment was associated with reversal of radiation-induced changes in linoleic acid metabolism and reduced levels of the lipid peroxidation marker 8-HDoHE (Figure 8). While these observations are consistent with reduced oxidative stress, the causal relationships between TPP-C6-NIT, Nrf2/GPX4, and metabolic changes remain to be determined. Molecular docking suggested a potential interaction between TPP-C6-NIT and Keap1 (Figure 3), providing a testable hypothesis for future mechanistic studies, but this computational prediction requires experimental validation.

Under identical experimental conditions, TPP-C6-NIT protected normal L-02 hepatocytes but not Huh-7 hepatocellular carcinoma cells (Figure 4). This selective cytoprotection is an interesting feature of TPP-C6-NIT. One speculation is that differences in mitochondrial membrane potential or baseline redox status between normal and cancer cells may influence drug accumulation or cellular response [33]. However, direct evidence is lacking, and the underlying mechanisms require further investigation. The lower or more heterogeneous mitochondrial membrane potential (ΔΨ_m_) in cancer cells may reduce TPP^+^-driven mitochondrial accumulation of TPP-C6-NIT, thereby diminishing its protective effect. Direct measurement of mitochondrial uptake in both cell types is needed to test this hypothesis. Nevertheless, this observation suggests that TPP-C6-NIT may have potential as a radioprotective adjuvant without compromising tumor cell killing, although this needs to be tested in tumor-bearing models.

To provide clarity on the strength of our evidence, we categorize our findings into three levels. First, robust functional findings: TPP-C6-NIT preserves mitochondrial structure and function (Complex III activity, ATP, ΔΨm, OCR) and reduces oxidative stress (ROS, MDA, 4-HNE, Fe^2+^) in vitro and in vivo (Figure 2, Figure 5 and Figure 6). Second, associated molecular correlates: TPP-C6-NIT treatment is associated with increased Nrf2 expression and restored GPX4 expression in irradiated liver (Figure 7), but direct evidence of Nrf2 nuclear translocation or transcriptional activation is lacking. Third, mechanistic hypotheses pending validation: molecular docking suggests potential interactions with Keap1 and Complex III (Figure 3), but these computational predictions require experimental validation (e.g., surface plasmon resonance, cellular thermal shift assays). This hierarchical presentation is intended to avoid overinterpretation of associative or computational data as mechanistic proof.

**Figure 10:**
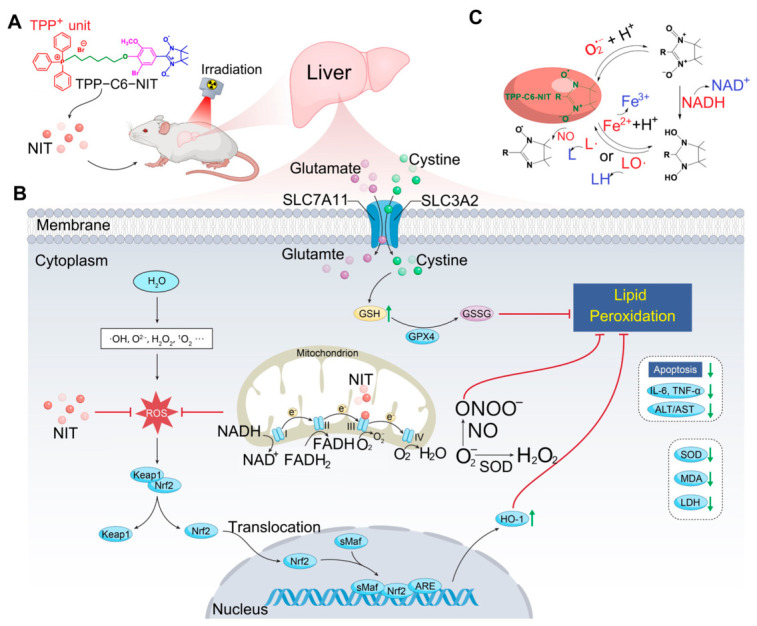
Schematic of TPP-C6-NIT’s multi-level protection against radiation-induced liver injury. (**A**) TPP-C6-NIT acts at mitochondrial, cellular, and systemic levels to alleviate radiation-induced liver injury in normal hepatocytes. (**B**) At the mitochondrial level, it scavenges radiation-induced ROS, preserves mitochondrial integrity, and is associated with increased Nrf2/GPX4 expression, reducing apoptosis and inflammation, thus protecting liver function. Its selective protection for normal cells over cancer cells widens the radiotherapy therapeutic window. (**C**) Redox transformation cycle of the nitronyl nitroxide moiety among nitroxide, hydroxylamine, and oxoammonium cation states.

Several limitations should be acknowledged. First, while we observed associations between TPP-C6-NIT treatment and changes in Nrf2/GPX4 expression, direct evidence of target engagement (e.g., Keap1 binding) or pathway activation (e.g., Nrf2 nuclear translocation) is not provided. Second, the metabolomics data are exploratory and do not establish causality. Third, the selective protection was observed in one hepatocellular carcinoma cell line (Huh-7) and was not tested in additional cancer types or in tumor-bearing animal models. Fourth, pharmacokinetics, biodistribution (including mitochondrial accumulation), long-term safety, and potential extrahepatic accumulation of TPP-C6-NIT remain to be evaluated. Finally, the molecular docking predictions are computational and require experimental validation. The proposed protective mechanisms of TPP-C6-NIT against RILI are summarized in Figure 10.

We acknowledge a major limitation regarding the animal model. The use of 6 Gy total body irradiation (TBI) does not replicate the clinical scenario of fractionated, partial-liver irradiation. The observed improvements in survival, weight loss, and peripheral cytopenias (Figure 5) suggest that TPP-C6-NIT provides systemic radioprotection, which likely contributes to survival beyond isolated liver protection. Therefore, our conclusions regarding liver protection should be interpreted within the context of acute systemic irradiation. Survival improvement may also involve protection of other radiosensitive organs (e.g., bone marrow, gut). Future studies using localized liver irradiation models are required to validate specific efficacy against clinical RILI.

## 5. Conclusions

In conclusion, TPP-C6-NIT is a mitochondria-targeted antioxidant that mitigates radiation-induced liver injury in normal hepatocytes and mice, while showing minimal protection in Huh-7 hepatocellular carcinoma cells. Its protective effects are associated with reduced oxidative stress, preserved mitochondrial function, and changes in Nrf2/GPX4 expression. This study provides a proof-of-concept for organelle-targeted radioprotection and identifies TPP-C6-NIT as a promising lead compound for further development. Future studies are needed to validate the proposed mechanisms and to evaluate its translational potential.

## Data Availability

The original contributions presented in this study are included in the article and Supplementary Materials. Further inquiries can be directed to the corresponding author.

## Supplementary Materials

The following supporting information can be downloaded at: https://www.mdpi.com/article/10.3390/antiox15060780/s1, Figure S1: Representative total ion chromatograms (TICs) of mouse liver tissue samples; Figure S2: Score plots from Principal Component Analysis (PCA); Figure S3: Orthogonal Projections to Latent Structures–Discriminant Analysis (OPLS-DA); Figure S4: Volcano plots of differential metabolites; Figure S5: Hierarchical clustering analysis of differential metabolites; Figure S6: Bubble chart of KEGG pathway enrichment analysis.

## Abbreviations

The following abbreviations are used in this manuscript:

IR	Ionizing radiation
ROS	Reactive oxygen species
WR2721	Amifostine
NIT	Nitronyl nitroxide radical
RBC	Red blood cell
WBC	White blood cell
HGB	Hemoglobin
PLT	Platelet
SOD	Superoxide dismutase
CAT	Catalase
MDA	Malondialdehyde
GSH-Px	Glutathione peroxidase
IL-1β	Interleukin-1β
TNF-α	Tumor necrosis factor-α
IL-4	Interleukin-4
H&E	Hematoxylin and eosin
