# Supplementary material for: A Mitochondria-Targeted Nitroxide Radical Mitigates Radiation-Induced Liver Injury by Attenuating Oxidative Stress and Preserving Mitochondrial Function
Source: Antioxidants (Basel). 2026 Jun 22;15(6):780. doi: 10.3390/antiox15060780 (PMC13295497; doi:10.3390/antiox15060780)
Supplement: Supplementary file 1 [file antioxidants-15-00780-s001.zip › antioxidants-4358866-supplementary.pdf]

Supplementary Information

A Mitochondria-Targeted Nitroxide Radical Mitigates Radiation-Induced Liver Injury by Attenuating Oxidative Stress and Preserving Mitochondrial Function

Miaomiao Li[1,2], Xiaojun Deng[1†], Haibo Wang[1*]

1. Department of Chemistry, School of Pharmacy, the Fourth Military Medical University, Xi'an, Shaanxi, 710032, China; 223040012830@email.sntcm.edu.cn (M.L.);
dengxiaojun@fmmu.edu.cn (X.D.)

2. Department of Pharmacy, Shaanxi University of Chinese Medicine, Xianyang 712046, China.

[*]Corresponding Author: haibo7691@fmmu.edu.cn

† These authors contributed equally to this work.

1. Untargeted Metabolomics Profiling of the Hepatic Protective Effect of TPP-C6-NIT

Untargeted metabolomics was performed to systemically investigate the metabolic alterations in mouse liver tissue induced by radiation and the intervention by TPP-C6-NIT. The high stability and reproducibility of the LC-MS analysis were confirmed by the tightly clustered quality control samples in the total ion chromatograms and principal component analysis (Figure S1, S2). Supervised OPLS-DA models revealed distinct metabolic profiles among the Control, IR, and IR+TPP-C6-NIT groups, demonstrating significant radiation-induced metabolic disturbances and a notable restorative trend upon TPP-C6-NIT treatment (Figure S3). We identified numerous significantly altered metabolites through volcano plot analysis (Figure S4). Hierarchical clustering of these differential metabolites further visualized the reversal effect of TPP-C6-NIT on the radiation-induced metabolic signature (Figure S5). Most importantly, KEGG pathway enrichment analysis pinpointed that TPP-C6-NIT intervention specifically reversed several key metabolic pathways disrupted by radiation, most notably glutathione metabolism and the ferroptosis pathway, thereby providing a systemic metabolic perspective for its protective mechanism (Figure S6).

A

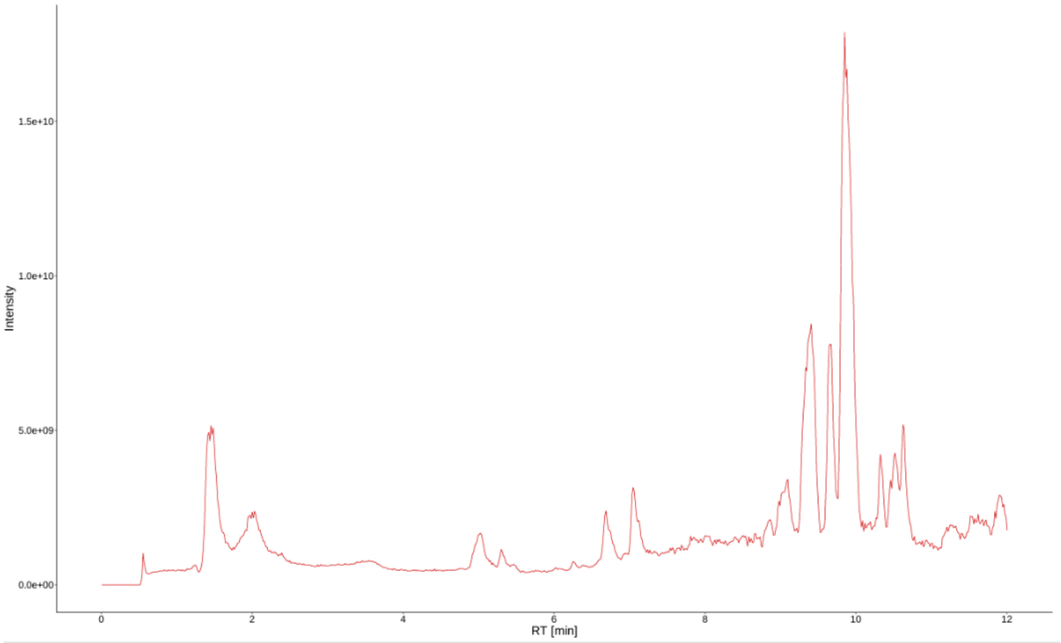

B

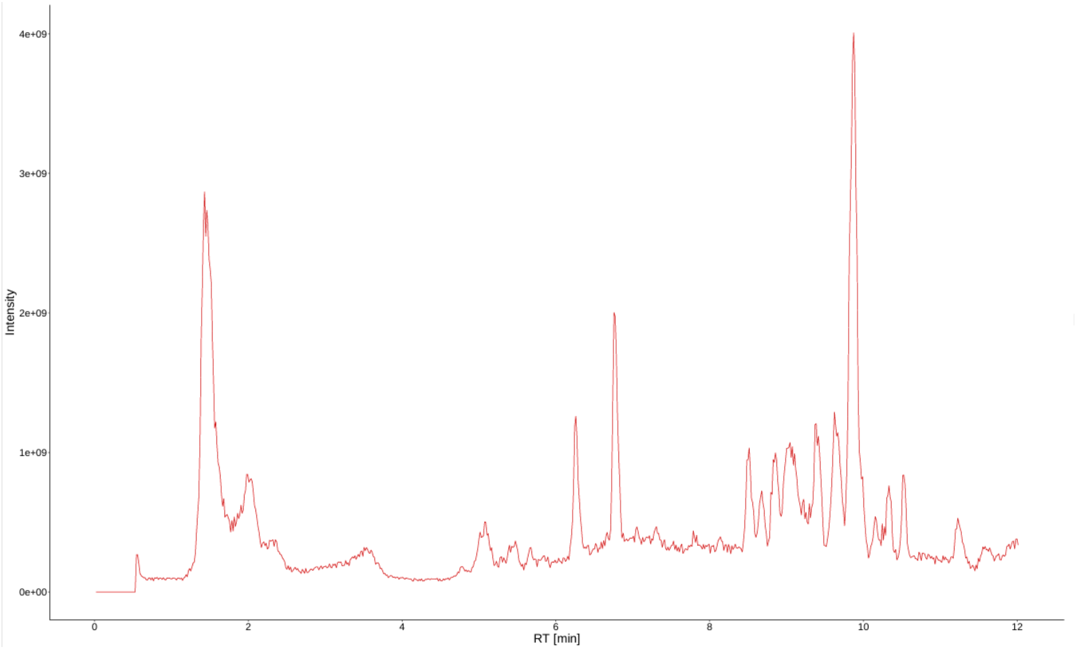

Figure S1. Representative total ion chromatograms (TIC) of mouse liver tissue samples acquired by LC-MS. **(A)** Positive ion mode; **(B)** Negative ion mode.

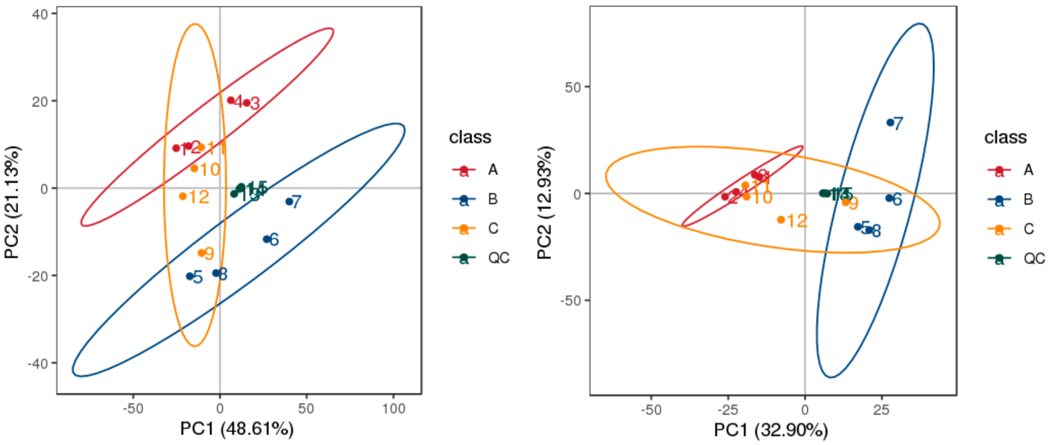

Figure S2. Score plots from Principal Component Analysis (PCA) of the metabolomics data. **(A)** Control group; **(B)** IR (Irradiation) group; **(C)** IR + TPP-C6-NIT treatment group.

A

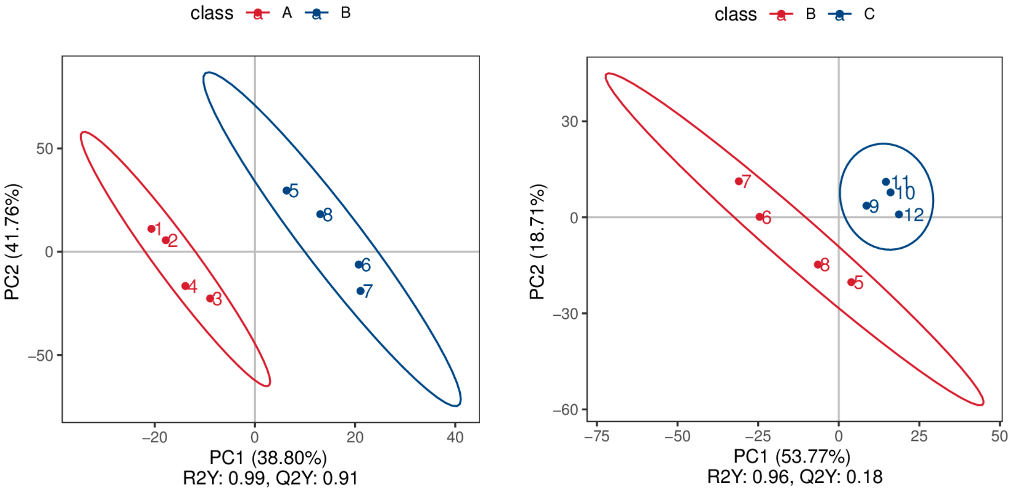

B

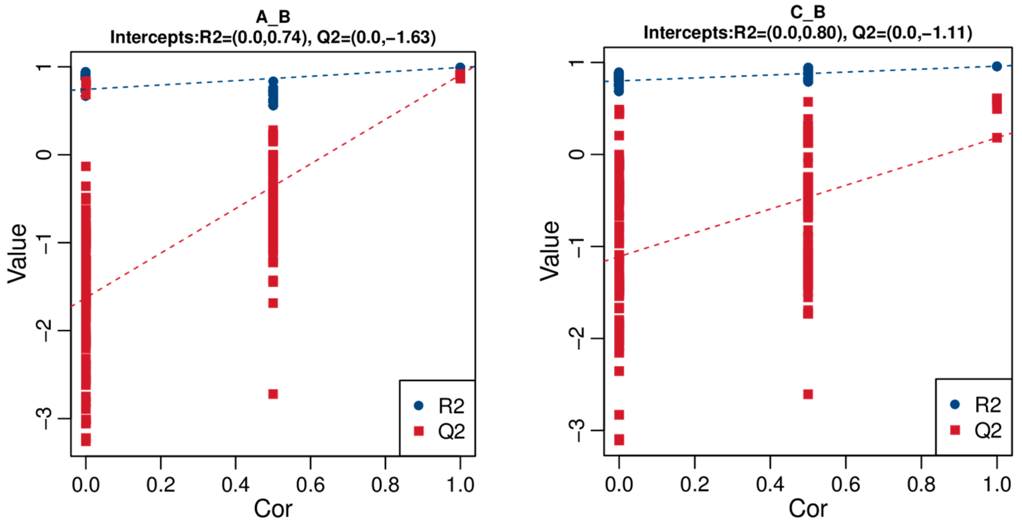

Figure S3. Orthogonal Projections to Latent Structures-Discriminant Analysis (OPLS-DA) of the liver tissue metabolite profiles from UPLC-Q-TOFMS. **(A)** OPLS-DA score plot in

negative ion mode; **(B)** Validation plot from 200 permutation tests for the OPLS-DA model in negative ion mode.

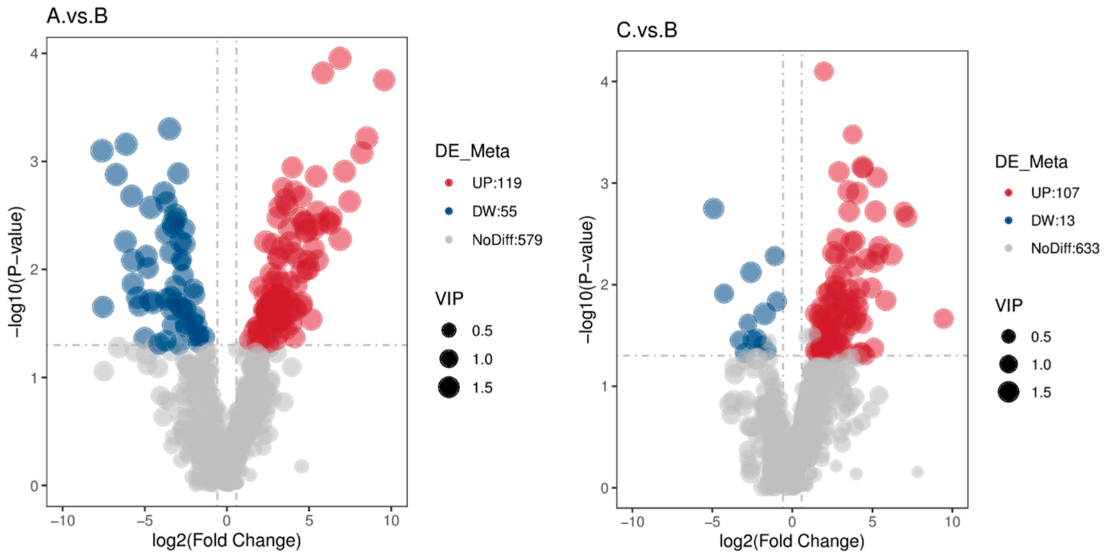

Figure S4. Volcano plots of differential metabolites. The x-axis represents the log_2-transformed fold change of metabolites between different groups, and the y-axis represents the statistical significance (-log_{10}(P-value)). Each point represents a metabolite. Metabolites with significant up-regulation are shown in red, and those with significant down-regulation are shown in blue. The size of the dots corresponds to the Variable Importance in Projection (VIP) value from the OPLS-DA model.

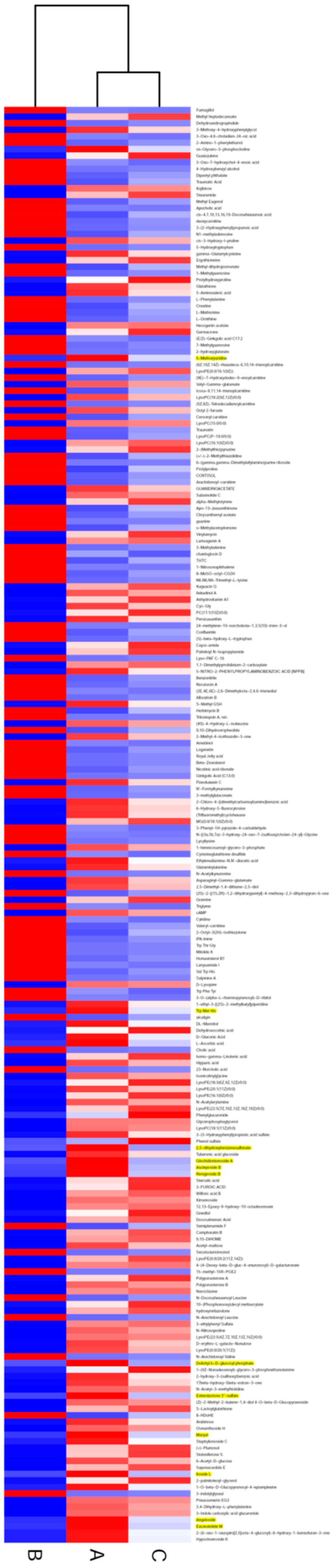

Figure S5. Hierarchical clustering analysis of differential metabolites. Rows represent metabolites, and columns represent samples from different groups (e.g., Control, IR, IR+TPP-

C6-NIT). The color scale represents the Z-score of the relative abundance of each metabolite, with red indicating high abundance and blue indicating low abundance.

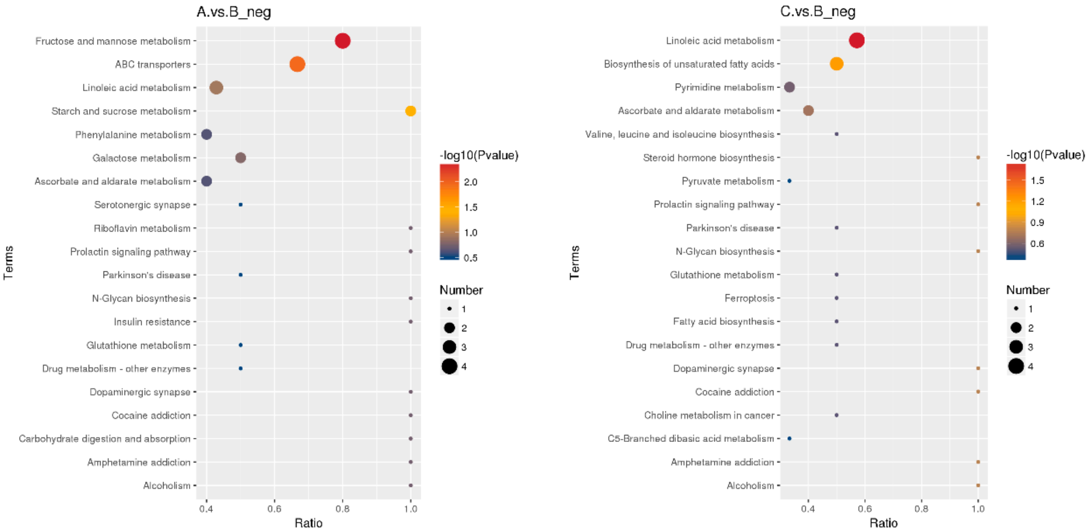

Figure S6. Bubble chart of KEGG pathway enrichment analysis for differential metabolites (Top 20 pathways are shown). The x-axis represents the Rich Factor (number of differential metabolites / total number of identified metabolites in that pathway). A higher Rich Factor indicates a greater degree of enrichment. The color of the bubbles represents the -$\log_{10}$(P-value) from the hypergeometric test, with a darker color indicating higher statistical significance. The size of the bubbles corresponds to the number of differential metabolites enriched in the pathway.
